# Empathy as a silent art–A doctor´s daily balancing act: A qualitative study of senior doctors’ experiences of empathy

**DOI:** 10.1371/journal.pone.0277474

**Published:** 2022-12-15

**Authors:** Johanna von Knorring, Arja Lehti, Martin Fahlström, Olof Semb

**Affiliations:** Unit of Professional Development, Department of Clinical Sciences, Umeå University, Umeå, Sweden; University of St Andrews, UNITED KINGDOM

## Abstract

Empathy in the doctor-patient relationship is of great importance and has long been considered a true professional virtue for doctors. Despite the general agreement concerning the importance of empathy, there is no consensus regarding the definition of empathy in medical research. While several quantitative studies, measuring empathy as an individual trait, show a decline in empathy among medical students, other studies have shown that empathy is influenced by contextual factors as well as the availability of role models. Therefore, further studies about the transition from medical school to clinical work also including the perspective of senior doctors are needed. The study presented in this article aims to better understand the clinical conditions for empathy through interviews with senior doctors about their lived experience of empathy. Twelve senior doctors, from different specialities were interviewed using a semi-structured approach. The data was analysed using content analysis. The analysis resulted in the main theme: Empathy as a silent art–a doctor`s daily balancing act. This main theme comprised three categories: “A tacit, yet language-dependent process”, “A daily balancing act” and “An unsupported path towards mastery”. Doctors face many challenges in their daily balancing act between individual and structural conditions that may affect empathy. In order to maintain and further develop empathy, doctors need working conditions allowing for collegial reflection and conversations that promote empathy.

## Introduction

Empathy in the doctor-patient relationship has long been considered a true professional virtue for doctors [1], highlighted in reflections on sustainable medical empathy [2]. Empathic engagement in patient care leads to increased communication and trust [3, 4] and contributes to improved clinical outcomes such as increased compliance and fewer complications [5–7]. Empathic engagement also protects against professional stress and burnout [8].

Despite a general agreement concerning the importance of empathy, there seems to be no consensus as to which definition of empathy in medical research is preferable [9, 10]. Neurobiological models include the activation of mirror neurons and complex neurobiological processes [9] while, in medicine, empathy is often primarily described as a cognitive trait, with emphasis on understanding the patient, communicating, and acting on that understanding [11]. Others highlight the affective aspects of empathy, focusing on the doctor’s ability to ‘feel with’ their patients [12]. Concern has been raised that focusing predominantly on cognitive aspects of empathy may promote a view that professional empathy does not benefit from emotional investment–a “detached concern”. In turn, this potentially eliminates the benefits of empathic engagement, making it more of a pastoral power than a true concern for what is best for the patient [13, 14].

Research has to a great extent focused on measuring empathy in medical students as they progress through their education. Many quantitative studies suggest that students’ empathy declines, particularly during the clinical years of their education [11, 15], while other researchers have questioned the results [16, 17]. Some of the research has also included residents and senior colleagues [15, 18].

To get a better overview of the transition from medical school to clinical work, we conducted a study among junior doctors in Sweden regarding their lived experience of empathy. The results were congruent with earlier research about the difficulties reaching a conceptual consensus. In that study, we concluded that empathy is contextual and influenced by parameters such as time restraints and workplace culture, and that the general understanding of empathy might be widened through the application of a socioecological model [19]. Additionally, we learnt that senior colleagues were particularly important as role models in developing and maintaining one’s empathic ability.

Based on this previous study, we saw the need for a better understanding of the corresponding lived experiences of senior doctors including their perceptions and experiences of empathy in clinical practice. The current study therefore aimed at understanding the clinical conditions for empathy by interviewing senior doctors about their lived experience of empathy.

## Methods

### Ethics

This study was approved by the Regional Ethics Review Board in Umeå, Sweden (Ref. No. 2016/50-31).

### Study design

Data was collected using semi-structured interviews (described below) and analysed through qualitative content analysis in order to convey the doctors´ lived experience while remaining close to the text [20].

### Setting and participants

This study was carried out in Sweden. Two university hospitals and one smaller, provincial hospital were approached assuming that the doctors have different professional experiences, including different responsibilities in the education of medical students.

Once the employer had approved the recruitment of doctors, interviewees were recruited trough a general email. Our goal was to recruit doctors from both surgical and non-surgical specialities, with longer and shorter interactions with their patients, in order to gain insight into different kinds of medical settings. Inclusion criteria were a minimum of five years´ experience as a senior doctor, having direct patient contact on a daily basis, and being able to conduct the interview in Swedish. Twelve doctors of different specialities chose to participate, ranging between 42–70 in years of age and with 8–32 years of experience as specialists. Four out of twelve identified as men and eight as women. Three worked in surgical and nine in non-operating specialities, such as Oncology and Internal Medicine. Three participants reported having official teaching assignment in medical education, while all participants were involved with medical students in their daily work.

### Data collection

The interviews were conducted between October 2018 and October 2019. Seven of the interviews were carried out by two of the authors (JVK and OS) and five by another experienced researcher. A semi-structured interview guide devised by three of the authors (JVK, AL, OS) was used to facilitate the interview and thereby achieve fuller content [21, 22]. Questions included: “Can you tell me about your experiences of empathy in your daily work?” and “How do you relate to being a role model for younger colleagues?”. Follow up questions were asked to obtain more detailed responses. The duration of the interviews was between 45–80 minutes.

### Data analysis

Interviews were recorded, transcribed verbatim and analysed using content analysis [20]. The analysis started with reading, re-reading, and identifying meaning units. The meaning units were then condensed and assigned codes close to the content. Codes were then organized into subcategories which, in turn, were sorted into categories forming the manifest content. An example of the coding is provided in Table 1 below.

**Table 1:** Example of the coding, meaning units and codes.

Meaning unit	Codes
***“I think the hardest thing* [regarding empathy] *is of course when the patient or their next of kin challenges your competence. That’s really difficult. I think everyone finds that.***	To be questioned is hard.
	To be challenged by patients or their relatives makes empathy difficult
***There was never any mention of empathy during my training. We didn’t even have a word for it.***	Empathy not being prioritized in training. Lack of language to talk about empathy.

These first steps were performed individually by two of the authors, JVK and AL, and then compared. Once there was consensus regarding the categories, three authors (JVK, AL and OS) re-read the transcripts together to formulate an overarching theme by interpreting the underlying meaning of the categories. This theme, along with the categories, formed the results of this study. An overview of the subcategories and categories can be found in Table 2.

**Table 2:** Overview of subcategories, categories, and the theme.

Subcategories	Categories	Theme
The changeable character of empathy.	A tacit, yet language dependent process	
Empathy is both wordless and language-dependent. Empathy is both person- and situation-dependent.		
Empathy is linked to obstacles and conflicts.	A daily balancing act	Empathy as a silent art–a doctor`s daily balancing act
A demanding process.		
Enabling and supporting factors.		
Empathy was fine-tuned as a result of greater professional experience.	An unsupported path towards mastery	
Impact of life experiences on empathy.		
Empathy as influenced by contextual change.		
The doctor as a role model.		
Conversations about empathy are difficult in the everyday clinical environment.		

After completing the first analysis, two participants were randomly selected and invited to review the preliminary results, as to reduce the impact of the authors’ prior assumptions and contextual understanding. The two participants found the material to be coherent with and, in general, expressing their experiences and no major changes were made.

## Results

Analysis of the data material revealed a main theme, that was given the name Empathy as a silent art–a doctor`s daily balancing act. The main theme includes three categories: “A tacit, yet language-dependent process”, “A daily balancing act” and “An unsupported path towards mastery”. The categories and their subcategories (in italics) are described below, the main theme will be further presented in the Discussion.

### A tacit, yet language-dependent process

The participants describe *the changeable character of empathy* as a quality that changes over time but also in different situations. The time aspect also entails relating to the patient both in the present and over time.

The participants differentiate between empathy and feeling pity, seeing this difference as both simple and obvious; they describe empathy not only as understanding the patient’s situation and perspective, but also acknowledging the patient in order to be able to act in their best interest.

Being able to deviate from treatment guidelines in order to better adapt care to the needs of the individual is described as empathic. Further, empathy might also entail refraining from treatment, if the doctor finds the treatment not to be beneficial to the patient–even if the decision goes against the patient’s wishes.

“*Even if there are treatment guidelines that prescribe a particular course of action and I see a patient with slightly different prerequisites*, *I adapt to the reality of the situation rather than follow the map blindly*. *And in my mind*, *empathy plays a part in this*.*” (P 9)*

The participants describe empathy as being wordless and embodied. Empathy is described as radiating confidence, and as the ability to accommodate the patient’s narrative. Nevertheless, it is evident how language influences empathy since asking follow-up questions is perceived as an empathic act.

The participants describe the importance of shared language, illustrated by examples of the uncertainty the doctor may experience as to whether or not empathy is conveyed whenever the consultation is dependent on an interpreter. They also highlight that an absence of verbal response represents an obstacle to empathy and that frustration on the part of the doctor or the patient makes communication, and therefore empathy, difficult.

While *empathy is both wordless and language-dependent*, it often takes a silent form, by means of body language, eye-contact, tone of voice and active listening.

“*You don’t always need to say that much*. *You present yourself as the person you are and the body you are*, *how you breathe*, *how you act*, *the patients read my body language…you have to be confident in acknowledging the person in front of you*, *and in doing so*, *you actually also expose yourself*.*” (P 11)*

The participants describe that *empathy is both person- and situation-dependent*, perceiving it as easier to empathize with people of similar background and with shared values. This leads to bias in terms of who incites empathy in us. For example, the respondents found it difficult to empathize with a patient with different explanatory models and expectations.

“*I think the hardest thing is when a patient or next of kin challenges your competence*. *When you actually know that this will not work out well*, *despite their wishes*. *And you try to explain but are treated with distrust*, *or with accusations of not understanding*, *or similar attitude*. *It is difficult to sustain empathy when someone directly contradicts my greater knowledge*.*” (P 8)*

Further, it is described as easier to feel empathy with severely or terminally ill patients even though, at the same time, it may be difficult to feel empathy with patients who cannot be cured. Certain medical conditions are strongly linked to lifestyle factors, such as smoking and obesity; respondents describe that it is harder to empathise with patients suffering from these conditions.

“*At times there is a lack of empathy in dealing with our patients due to a perception that they are a bit undeserving*. *If they had just exercised and eaten properly*, *you wouldn’t have to operate on them in the first place*, *and then we could operate on more cancer patients and not have a waiting list*. *… Or the psychosomatic patients we have*. *Even though I try to understand that they are suffering*, *sometimes I think they should just pull themselves together*.*” (P 6)*

### A daily balancing act

The participants describe that *empathy is linked to obstacles and conflict* in patient encounters. The production requirements for care impact negatively on empathic capacity both short and long term. Conflicts arise when the health care programme is inconsistent with individual needs but also if the doctor’s decision is disputed, in the latter case, such as when exercising mandatory use of authority prescribed by law, or when discussing to what care the patient is entitled. Empathy becomes obstructed by patients’ negative emotions, sometimes made more difficult by the presence of family members. The person behind the diagnosis might not be attended to in the encounter while at the same time working under stress and time pressure.

Empathy is described as a *demanding process* that requires effort in every patient consultation, and effort in order to be sustained over time. However, empathy is also perceived as a motivating factor for coping and experiencing meaningfulness. Whenever the participants experience difficulty accessing empathy or find it to be diminished, they describe protective strategies to avoid becoming emotionally burdened, for example, withdrawing mentally, not asking questions during the consultation, or conducting the consultation on autopilot–all of which is found to be detrimental to empathy.

“*You have to regulate how sensitive you are*. *It’s difficult to maintain because it does of course form the basis of the encounter*, *but you can’t get in too deep*. *It is a tricky balancing act*. *A lot of people deal with a difficult situation by not getting involved*, *by keeping a distance*, *while others get in too deep and get caught up in it*, *which affects their ability to cope with the situation*.*” (P 10)*

There are many daily challenges that may negatively affect an empathic approach. The participants portray the struggle to achieve balance in exercising empathy as a daily balancing act between the best interests of the individual patient and a sort of benefit principle for all patients. They must balance the number of encounters with the quality of each encounter. Means and time have to be allocated to each individual patient based on the finite resources available within the healthcare system. There is also need for balance between discerning the patient’s needs and situation, and the doctor’s agenda in the encounter.

The daily balancing act also involves various *enabling and supporting factors*. The participants describe curiosity and active presence in the here-and-now during the consultation as important and enabling empathy. Acquiring an understanding of one’s working colleagues through teamwork is seen as helpful in maintaining an empathic approach. It is also described as helpful should the doctor experience a temporarily diminished capacity for empathy, as colleagues will pick up the slack.

“*To be present*, *that’s the sort of person I am*. *I’m a very mindful person and I can deal with difficult things in quite a short time*. *It’s time efficient*. *But if you’re not like that or if you haven´t thought these questions through*, *then you might choose to shield yourself instead*. *Which actually becomes less time-efficient*, *plus it can probably cause you to burn out*.*” (P 9)*

Support from colleagues is described as important when discussing difficult decisions and reactions in patient encounters. Nurses are described as more accustomed to discussing empathy and may provide better support than doctors. However, it may be just as important to confide in a friend who is completely outside the health service in order to gain new perspectives. The participants also express need for more professional support, e.g. from psychologists, in order to better understand difficult professional situations and improve self-awareness, which they associate with improved empathy.

Resources were allocated for supportive discussions about empathy early in their career, but formal forums are not available to trained specialists. Regarding personal strategies for maintaining and developing empathy, the participants consider recovery to be very important. This can take a number of forms, such as performing or listening to music or attending cultural events or spending time outdoors and in the countryside.

“*There’s no room for reflection*. *You may feel that things didn’t work out well with a patient*, *and you could usually do better*, *but why*? *Maybe I wasn’t able to give 100 percent*, *but the relation and the encounter could have been better*. *In any case*, *there just isn’t time for that kind of reflection in my working day*.*” (P 10)*

“*I think we are expected to be good enough at it*. *I don’t think it’s something than can be measured*, *and as a result*, *in the modern healthcare system it isn’t prioritised*. *We used to have a structured discussion group where*, *at first*, *a psychologist led the session*, *and then we were supposed to lead the sessions ourselves*. *Issues relating to empathy could still crop up here*, *but it eventually ran out of steam*.*” (P 12)*

### An unsupported path towards mastery

Participants describe a complex change in which *empathy was fine-tuned as a result of greater professional experience*. They describe a shift during training, from identifying with the patient to identifying with the role of doctor, as something that makes empathy easier.

“*In some way it was the first step towards professionalisation*, *when we go into a room now*, *I automatically see myself as one of the doctors rather than as one of the potential patients*.*” (P 6)*

They describe their experience as having contributed to an acceptance that not everyone can be happy, and that not everyone can be helped–something that used to cause frustration. No longer as affected, sad or preoccupied when faced with a certain procedure or disease, they simultaneously describe the risk of running on autopilot–something they try to prevent by maintaining a genuine curiosity in every encounter. At the same time, they describe their level of experience as something that permits them to be more affected and more present in the encounter. Their increased professional confidence allows a bird’s-eye view of the encounter, where previously they would have focused on medical-technical aspects. Greater confidence also contributed to an understanding of empathy as being situation-dependent and therefore needing to be adapted, both between different encounters and at each stage of the encounter.

“*I lacked experience and saw myself as ignorant*. *Talking to patients weighed on me more then*. *I found it difficult to let go*, *I got really affected and sometimes I had a hard time ending conversations*. *Over time*, *I have become more confident*, *both as a doctor and in my conversations with the patient*. *That has much to do with experience*, *and indeed with life*.*” (P 7)*

The *impact of life experience on empathy* is also described, appearing to contribute to a better understanding of the situations of others which, in turn, facilitates an all-inclusive perspective on patients and colleagues. Aspects of your own life influence how much of an impact a patient will make, and how difficult it is to let go of them at the end of the workday.

“*It has occurred to me*, *as a doctor*, *that it is the patients closest in age to you or to your children that you most often have an emotional reaction to*. *And of course*, *it changes with age*. *Now it’s suddenly severely ill 45-year-olds with children who are the hardest encounters*, *because they remind me of myself*.” *(P 10*, *P 1)*

They also describe *empathy as influenced by contextual changes*. For example, patients’ expectations have changed, with increased demands for shared explanations and solutions, requiring empathic engagement. Also, students are described as more individualistic and with other values compared to earlier generations, which makes it harder to adopt an empathic approach in supervising them.

Concerning *the doctor’s role as a role model*, the participants describe there being few role models in their early years. And they were mostly admired from a distance. It was therefore difficult to start conversations about encounters, empathy and development in these areas. The participants now see that they function as such, but whether or not they are good role models is difficult to ascertain. They express concern that there is not enough time to mentor today’s medical students and young clinicians, making it difficult to be a good role model in day-to-day work.

“*There was no talk of empathy during my training*. *We didn’t really even have a word for it*, *there were only a few colleagues who really knew how to look after patients and who became empathic examples*. *I don’t think we really had any training at all in this during our education*.*” (P 3)*

Participants describe that a culture can be created that promotes or hinders conversations about empathy but *conversations about empathy are difficult in the everyday clinical environment*. They describe a lack of words for empathy, meaning it is only indirectly noticeable in discussions and jargon concerning patients among colleagues–even if the culture promotes empathy. Lacking official forums, there are too few conversations about empathy, patient encounters and difficult meetings. For this reason, discussions about encounters or the absence of an empathic approach only take place when something goes wrong.

“*What I’m trying to say is that you should also work in a structured way with these issues*. *But it is difficult to work in a structured way with abstract matters*.” (P 10)

## Discussion

**Fig 1:**
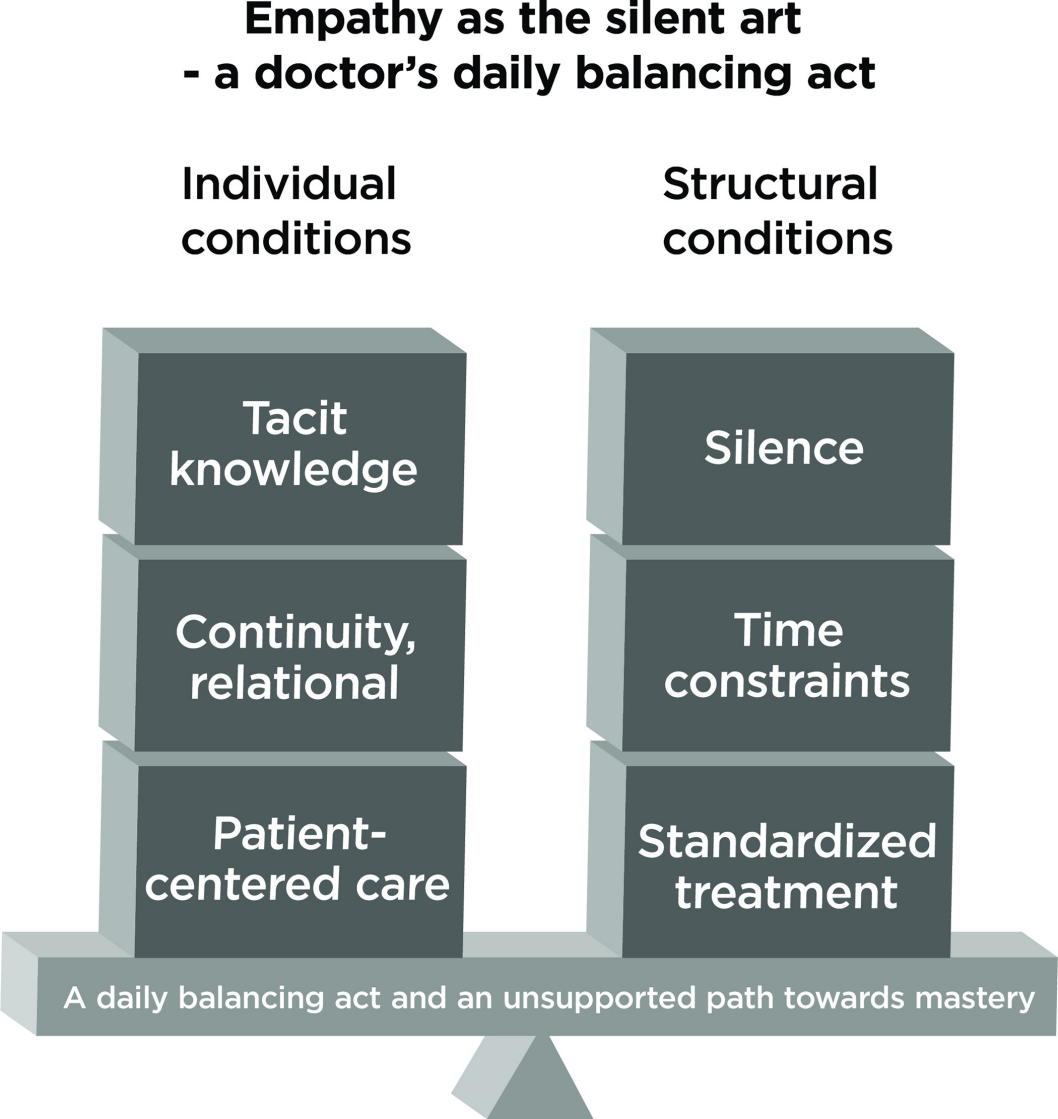
A model illustrating the main theme “Empathy as a silent art—a doctor’s daily balancing act.

The aim of this study was to further understand the clinical conditions of empathy. To the senior doctors in this study, empathy in the daily clinical practice is a difficult balancing act. We have chosen to discuss this from individual as well as structural conditions (see Fig 1) to show how the balancing act might undermine empathy in the doctor-patient relationship and how it complicates role modelling empathy to younger colleagues, constituting an unsupported path towards mastery.

On an individual level, the doctors experience a lack of training in talking about, as well as the necessary concepts required for conversation about, empathy. Also, since they experience empathy very much as a non-verbal phenomenon, describing how empathy is performed is difficult [23]. On a structural level, the lack of a shared language makes it difficult to assess empathy in colleagues as well as provide necessary feedback. No forums or physical locales are provided where these conversations can take place, although doctors need allotted time and opportunities for reflection on practice, in-depth studies as well as educational networks provided by the employer [24]. This is important for the continuing professional development as well as the working conditions and well-being of doctors [25]. Empathy therefore remains a tacit knowledge, i.e. the phenomenon where a practitioner knows how to do something without being able to express or describe the explicit knowledge behind a certain skill [26]. Tacit knowledge is made conscious, in part, through reflection, but due to the lack of opportunities doctors have not been able to transform empathy into something that can be verbalized, much less taught to others.

The second part of the balancing act is that between empathy as something that requires both relational effort and continuity, and the time restraints of the doctors’ daily schedule. The senior doctors describe that it takes time to build a relational foundation with the patient, where trust and commitment to treatment develops gradually, but this is often impaired by how the health care system functions on a structural level. They raise critique against short time slots per patient and little or no time to reflect in between patients. Further, they describe the lack of continuity as the major obstacle to empathic encounters and sustainable relationships. These findings add to earlier research on the negative impact of a stressful work environment on empathy [27].

The third part describes the daily balancing act between patient-centred care and standardized treatment. They describe a challenging process where they must evaluate the needs of each patient in juxtaposition to standardised care plans and the needs of the many. This is not surprising and to some extent necessary to provide good and equal care, but the conflict of interest seems to consume time and energy, the consequences being a conflicted conscience and stressed doctors. Further, they describe a clash between their personal beliefs and a working culture prioritizing medical knowledge and technique rather than empathy and patient-centred care.

In contrast with the studies showing a decline in medical students’ empathy [10], empathy, for senior doctors, appears to be of importance and present in their everyday working life. Unfortunately, structural problems such as a stressful work environment and the short time allotted for each patient makes little room for empathy both during individual consultations and in the maintenance and development of empathy. Previous research has shown junior doctors’ need for better role models [19], but senior doctors consider their own ability and development in that respect as lacking. The doctors describe their own journey from being young physicians looking up to distant role models, to having the title of role model thrust upon them without knowing how to go about it or what constitutes a good role model, lacking formal training in how to be one. The work-place culture allows neither time enough together with students, nor any purposeful training. Instead, being a role model becomes a matter of trial and error. Doctors also perceive that they are expected to be independent due to their many years of experience while they still need to discuss their own personal and professional development.

It is evident that, with the absence of interest in empathy on a structural level, it becomes the concern of the individual doctor to create opportunities for conversations about empathy, as well as other important issues like being a role model and promoting a culture for continuous learning and development. Similarly, recreation and recovery are left in the hands of the individual doctor. Strategies for stress management and recovery is a matter of personal initiative, independent of the workplace, as it is consigned to the doctor’s spare time.

In our view, the results of this study highlight what may be a shift in perspective on empathy. In order for doctors to maintain and develop empathy, the health care system and policy makers need to improve structural conditions rather than transferring the responsibility to the already encumbered doctors.

### Strengths and limitations

This study focuses on 12 senior doctors’ views and experiences about empathy in Sweden, which makes the results influenced by contextual aspects of both empathy and the Swedish health care system. The participants represent variations in age, medical specialities and work experience, as well as sizes of hospitals and geographical location, which increases the transferability to other medical contexts [20].

All of the authors have different backgrounds (Clinical Psychology, Family Medicine, Rehabilitation Medicine and Oncology). These different backgrounds widened the collective perspective on the data analysis. Three of the authors have working experience as doctors. This made it easier to understand the context and situations described by the participants. On the other hand, this pre-existing knowledge might increase the risk of overlooking important findings that could benefit from further investigation. One of the authors is a psychologist who contributed with an outsider perspective. During the whole research process, the authors strived to remain aware of the interaction between the pre-existing knowledge of the researchers and the findings through continuous discussion. Further, two of the informants read and confirmed the results, which adds to the credibility of the results [20].

A scope for future research could be to investigate various interventions in the working environment that promote empathy and role modelling, assessed through both qualitative and quantitative methods.

## Conclusions

Doctors face many challenges in their daily balancing act between individual and structural conditions that may affect empathy. In order to develop and maintain empathy, doctors need improved working conditions allowing for collegial reflection and conversations that promote empathy.
